# Tie2-Expressing Monocytes Are Associated with Identification and Prognoses of Hepatitis B Virus Related Hepatocellular Carcinoma after Resection

**DOI:** 10.1371/journal.pone.0143657

**Published:** 2015-11-23

**Authors:** Yi-Feng He, Chao-Qun Wang, Yao Yu, Jing Qian, Kang Song, Qi-Man Sun, Jian Zhou

**Affiliations:** 1 Department of Liver Surgery, Liver Cancer Institute, Zhongshan Hospital, Fudan University, Shanghai, China; 2 Key Laboratory of Carcinogenesis and Cancer Invasion of Ministry of Education, Shanghai, China; 3 Department of Immunology, Shanghai Medical College, Fudan University, Shanghai, China; 4 Institutes of Biomedical Sciences, Fudan University, Shanghai, China; National Health Research Institutes, TAIWAN

## Abstract

**Background:**

Tie2-expressing monocytes (TEMs) are found in various tumors, involved in forming tumor blood vessels and expressing several important proangiogenic factors. The goals of this study were to evaluate the value of TEMs in diagnosing and predicting the prognosis of hepatitis B virus (HBV)-related hepatocellular carcinoma (HCC).

**Methods:**

Flow cytometry was performed to identify and count TEMs in peripheral blood monocytes from HCC patients (n = 84) receiving hepatectomy, HBV cirrhotic patients (n = 21), benign tumors patients (n = 15) and healthy volunteers (n = 23). Angiopoietin-2 (Ang-2) levels in the plasma were determined by enzyme linked immunosorbent assay. The distribution of TEMs in tumor tissue was observed by immunofluorescence staining. Then we determined the vascular area as a percentage of tumor area (vascular area/tumor area) by immunohistochemical staining. Finally the prognostic significance of TEMs and other clinicopathologic factors was evaluated.

**Results:**

Percentage of TEMs in peripheral blood monocytes significantly increased in HCC patients compared with HBV cirrhotic patients and healthy donors (both *P*< 0.001). However there was no significance in benign liver tumor (*P* = 0.482). In addition, the percentage of circulating TEMs was positively correlated with plasma Ang-2 concentration (*P*<0.001, r^2^ = 0.294) and vascular area/tumor area (*P*<0.001, r^2^ = 0.126). Furthermore the percentage of intratumoral TEMs was significantly higher than that of paratumoral TEMs (*P*<0.001). Increased circulating TEMs was associated with poor overall survival (*P* = 0.043) and a shorter time to recurrence (*P* = 0.041). Multivariate Cox analysis also revealed that the percentage of TEMs in peripheral blood was an independent factor for HCC patients’ prognosis.

**Conclusions:**

TEMs may promote angiogenesis in HCC regarding the angiopoietin/Tie2 signal pathway. Percentage of TEMs in peripheral blood monocytes may be applied as a biomarker for identifying HBV-related HCC and predicting the prognosis of these patients after resection.

## Introduction

Hepatocellular carcinoma (HCC) is one of the most common malignant tumors in the world. Most cases of HCC are secondary to hepatitis B virus (HBV) infection in China. The 5-year survival of HCC patients in all stages is less than 16% [1]. So investigation of predictive factors for prognosis will be useful for developing better strategies for HCC treatment. HCC is also one of the most abundant vascular solid tumors, in the formation and growth of which angiogenesis plays an important role. In 2005, De Palma [2] discovered a new subset of monocytes expressing the tyrosine kinase receptor Tie2 (tyrosine kinase with immunoglobulin and epidermal growth factor homology domains 2) as a representative surface marker. Tie2-expressing monocytes (TEMs) have been found in various human tumors, to form tumor blood vessels and express several proangiogenic factors such as basic fibroblast growth factor (b-FGF), vascular endothelial growth factor (VEGF), and matrix metalloproteinase-9 (MMP-9) [3–5].

Angiopoietin-2 (Ang-2) was originally regarded as a specific ligand of Tie2 [6]. The significance of Ang-2/Tie2 pathway in neovascularization had been clarified before [7]. In the circumstances of hypoxia, endothelial cells could be activated and secrete Ang-2 from Weibel-Palade bodies [8], recruiting TEMs to tumor site where they stimulated angiogenesis [9].

In view of the importance of TEMs in tumor angiogenesis and the vascular-rich character in HCC associated with poor prognosis, we investigated whether TEMs are present in HBV-related HCC and its role in diagnosing, predicting the prognosis, and future application to treatment.

## Materials and Methods

### Ethics Statement

The analysis of blood samples and tissues was approved by the Research Ethics Committee of Zhongshan Hospital, Fudan University. All the participants provided their written informed consent to participate in this study.

### Patients and Specimens

HCC patients with HBV infection (n = 84) receiving hepatectomy from February, 2012 to September, 2012 were included in the study. The inclusion and exclusion criteria of patients and treatment modalities have been described previously [10]. Pathological diagnosis was determined through the histology of tumor specimens. Clinical stage of HCC was decided by the Barcelona Clinic Liver Cancer Group (BCLC) classification system [11]. The patients with HBV-related liver cirrhosis (n = 21), benign liver tumors (n = 15) and healthy volunteers (n = 23) were also enrolled as controls. The pathological diagnoses of benign tumors are as follows: 8 cases were hemangioma, 6 cases were hepatic angioleiomyolipoma and 1 case was adenoma. Blood samples from patients were collected prior to surgery. Patient characteristics and disease classification are shown in Table 1.

**Table 1:** Clinical characteristics of research participants.

Clinicopathologic characteristics	HCC	LC	BLT	HD
**Number of patients**	84	21	15	23
**Gender (male/ female)**	76/8	14/7	6/9	13/10
**Median age (yr)**	54.5	57.0	44.0	61.3
**Child-Pugh grade(A/B/C)**	79/5/0	0/12/9	-	-
**HBVDNA, Copies/ml (<10** ^**3**^ **/>10** ^**3**^ **)**	58/26	9/12	-	-
**AFP, ng/ml (≤20 />20)**	40/44	17/4	-	-
**Edmonson grading(I/II/III/IV)**	2/49/31/2	-	-	-
**Tumor size, cm (≤5 />5)**	57/27	-	-	-
**Tumor number(single/multiple)**	65/19	-	-	-
**Microscopic Vascular invasion(present/absent)**	34/50	-	-	-
**Macroscopic Vascular invasion(present/absent)**	2/82	-	-	-
**BCLC stage (0/A/B/C)**	15/45/18/6	-	-	-

Abbreviations: BCLC, Barcelona-Clinic Liver Cancer Group; HCC, hepatocellular carcinoma; LC, liver cirrhosis; BLT, benign liver tumor; HD, healthy donors

### Follow-up

All the patients were closely observed until June 1^st^, 2015. The regular follow-up protocol included liver function, serum alpha-fetoprotein (AFP) and abdominal ultrasonography at intervals of 2 to 3 months. CT/MRI scan was then performed every 6 months. Extrahepatic organ examination would be carried out if patients were doubted to have metastasis. Overall survival (OS) was specified as the interval between the dates of surgery and death. Recurrence-free survival (RFS) was calculated as the interval between the dates of operation and recurrence. Patients without recurrence or death were censored at the last follow-up. The diagnosis of recurrence was according to pathological evidence or typical image appearance. Patients with confirmed recurrence were given further treatment, such as hepatectomy, transcatheter arterial chemoembolization or radiofrequency ablation.

### Flow Cytometric Analysis

**Fig 1:**
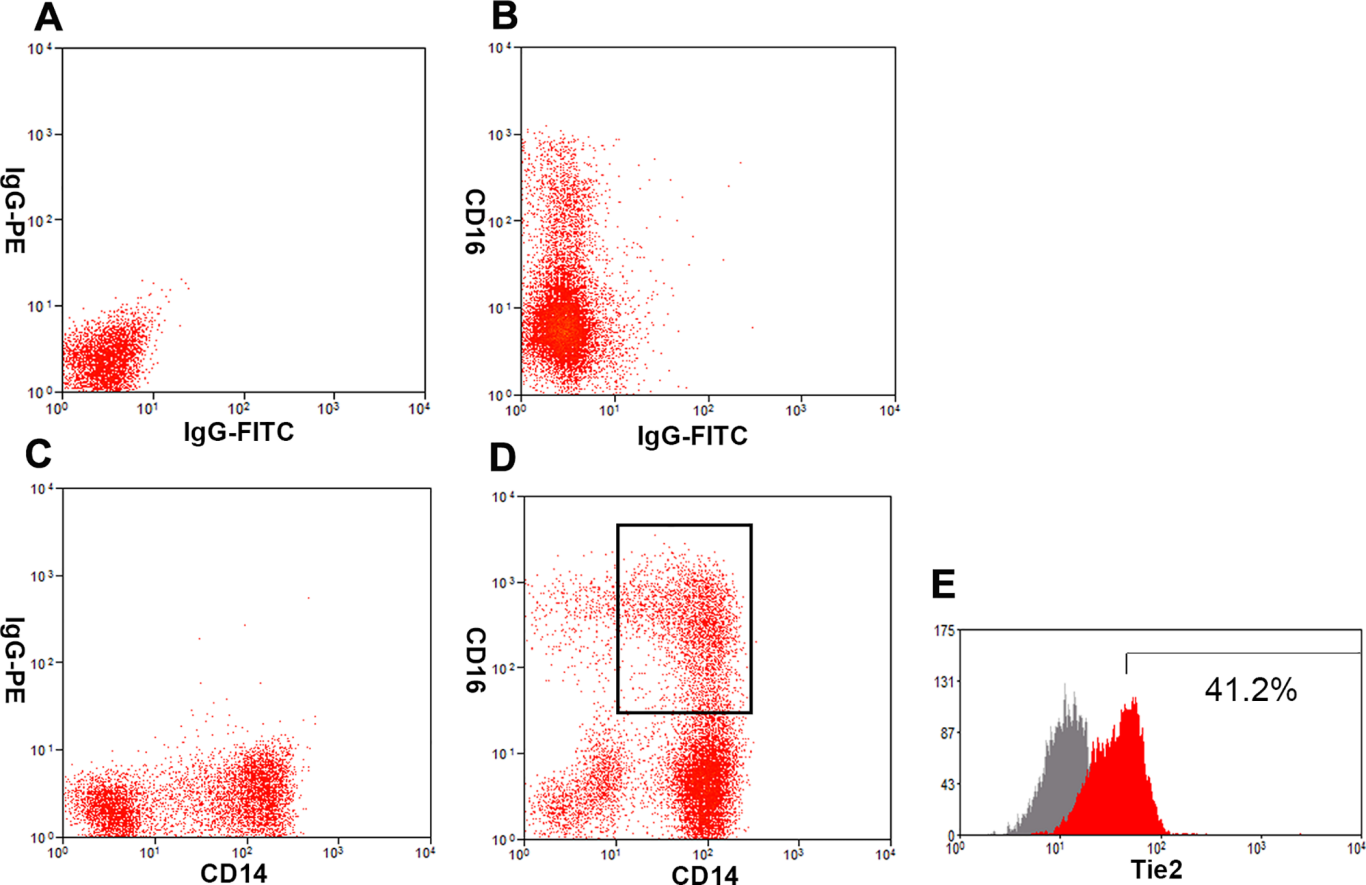
Expression of Tie2 on peripheral blood CD14^+^CD16^+^ monocytes. (A) Purified peripheral blood monocytes were stained with conjugated control antibodies (IgG-FITC, IgG-PE) or with CD16-PE antibody alone (B) or with CD14-FITC antibody alone (C) to determine the presence of CD14^+^CD16^+^ monocytes (D). (E) TEMs were distinguished by using IgG-APC control and Tie2-APC antibodies. The percentage of TEMs in peripheral blood CD14^+^CD16^+^ monocytes for this representative sample was 41.2%.

Peripheral blood mononuclear cells (PBMC) were isolated from 10mL of whole blood by Ficoll density centrifugation. After PBMC were labeled with a mouse anti-human FITC-conjugated CD14 antibody (557153, BD Bioscience, San Jose, CA, USA), a mouse anti-human PE-conjugated CD16 antibody (556619, BD Bioscience, San Jose, CA, USA) and a mouse anti-human APC-conjugated Tie2 antibody (FAB3131A, R&D System, Minneapolis, MN, USA), flow cytometry was carried out on a Becton Dickinson FACS Calibur (Beckmam Coulter Inc., Brea, CA, USA) to seek TEMs defined as CD14^+^CD16^+^TIE2^+^ cells [12]. Summit software version 4.3 (Beckmam Coulter Inc., Brea, CA, USA) was utilized to analyze FACS-data. Isotype-matched antibodies were applied with all the samples as controls (Fig 1).

### Ang-2 measurement by enzyme linked immunosorbent assay

Plasma obtained from an EDTA-anticoagulated blood sample was kept at −80°C. Ang-2 levels in the plasma were acquired by enzyme linked immunosorbent assay **(**ELISA) using the avidin-streptavidin method. Absorbance was recorded at 495 nm, and Ang-2 concentrations were read from the standard curve.

### Immunofluorescence staining

Immunofluorescence staining (IFS) was done as previously described with some modifications [3]. Briefly, samples obtained from surgical resection were snap-frozen. Four-micrometer sections were fixed in 4% paraformaldehyde for 15 minutes and blocked by 5% bovine serum albumin (BSA). Then sections were incubated with rabbit anti-human CD14 antibody (sc-9150, Santa Cruz Biotechnology, CA, USA) and goat anti-human Tie2 antibody (sc-31266, Santa Cruz Biotechnology, CA, USA) or mouse anti-human CD16 antibody (sc-20052, Santa Cruz, Santa Cruz Biotechnology, CA, USA). Alexa Fluor 488-labeled goat anti-rabbit IgG and Alexa Fluor 594-conjugated donkey anti-goat or goat anti-mouse IgG (Invitrogen, Karlsruhe, Germany) were applied as secondary antibodies to mark tissue-bound anti-CD14 and anti-Tie2 or anti-CD16, respectively. Sections were imaged on fluorescence microscope (LEICA, Wetzlar, Germany).

### Vascular area as a percentage of tumor area

Paraffin embedded tumor blocks were cut (4μm sections) and mounted on adhesive slides. Vessels were detected by using immunohistochemistry with CD34 [13]. To determine the vascular area as a percentage of tumor area (vascular area/ tumor area), the stained sections were firstly screened microscopically at low power (X50) to find the areas of highest vascularization. Then five intratumoral high power (X100) fields were selected randomly and vascular area in each high power field was calculated by Image-Pro Plus software (Media Cybernetics Inc., Rockville, MD). The vessel area/tumor area was calculated as the mean value of five regions of interest.

### Statistical analysis

Statistical analysis was calculated with SPSS 13.0 (SPSS Inc., Chicago, IL, USA). Values are presented as the median or mean ± standard deviation. Differences between two groups were computed by the Mann-Whitney nonparametric U test, and multiple comparisons between more than two groups by the Kruskal-Wallis nonparametric test. Cut off value of TEMs percentage for survival prediction was determined by receiver operating characteristic (ROC) analysis. The OS and RFS rate in HCC patients after hepatectomy was estimated by Kaplan–Meier method, with the Log-Rank test for comparison. The Cox proportional hazard model was utilized for multivariate estimation and variables associated with prognoses by univariate analysis were regarded as covariates. If *P* value was less than 0.05, the difference was considered statistically significant.

## Results

### Percentage of TEMs in peripheral blood monocytes in different group

**Fig 2:**
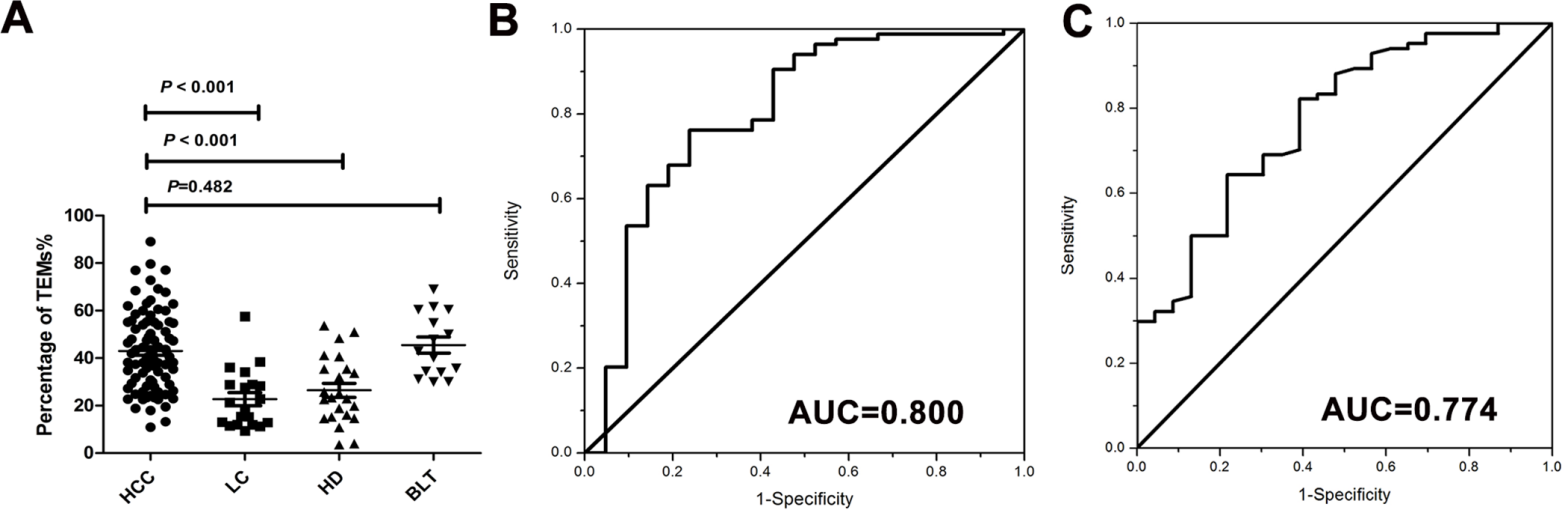
Percentage of TEMs in peripheral blood monocytes from different groups and diagnostic value of TEMs. **(A)** The *P* value was calculated by Mann-Whitney nonparametric U test. HCC, hepatocellular carcinoma; LC, liver cirrhosis; HD, healthy donors; BLT, benign liver tumor. **(B)** ROC curve analysis of TEMs value in diagnosing HCC from liver cirrhosis. The optimal cut off value was 28.9%, and the sensitivity and specificity were 76.2% and 76.2% respectively, with an area under the curve of 0.800 (95% CI, 0.704–0.896, *P* < 0.001). **(C)** ROC curve analysis of TEMs value in diagnosing HCC from healthy donors. The cut off value of 26.2% yielded the best sensitivity (81.0%) and specificity (60.9%) with the area under the curve of 0.774(95% CI, 0.670–0.878, *P* < 0.001).

Circulating TEMs were detectable in all the subjects. For HCC patients, the percentage of TEMs in peripheral blood monocytes (median: 41.2%) was significantly elevated compared with that in patients with HBV-related liver cirrhosis (median: 21.3%, *P* < 0.001) and healthy donors (median: 23.4%, *P* < 0.001; Fig 2A), however there was no statistical significance with benign liver tumor (median: 42.9%, *P* = 0.482; Fig 2A). For the groups of HCC patients and patients with HBV-related liver cirrhosis, the optimal cut off value of the percentage of TEMs was 28.9%. The sensitivity and specificity were 76.2% and 76.2% respectively, with an area under the ROC of 0.800 (95% CI, 0.704–0.896, *P* < 0.001; Fig 2B). ROC curve also indicated that the cut off value of 26.2% yielded the best sensitivity (81.0%) and specificity (60.9%) for differentiating HCC patients from healthy volunteers. The area under the ROC curve was 0.774(95% CI, 0.670–0.878, *P* < 0.001; Fig 2C).

### Plasma Ang-2 concentration was positively correlated with percentage of TEMs in peripheral blood monocytes

**Fig 3:**
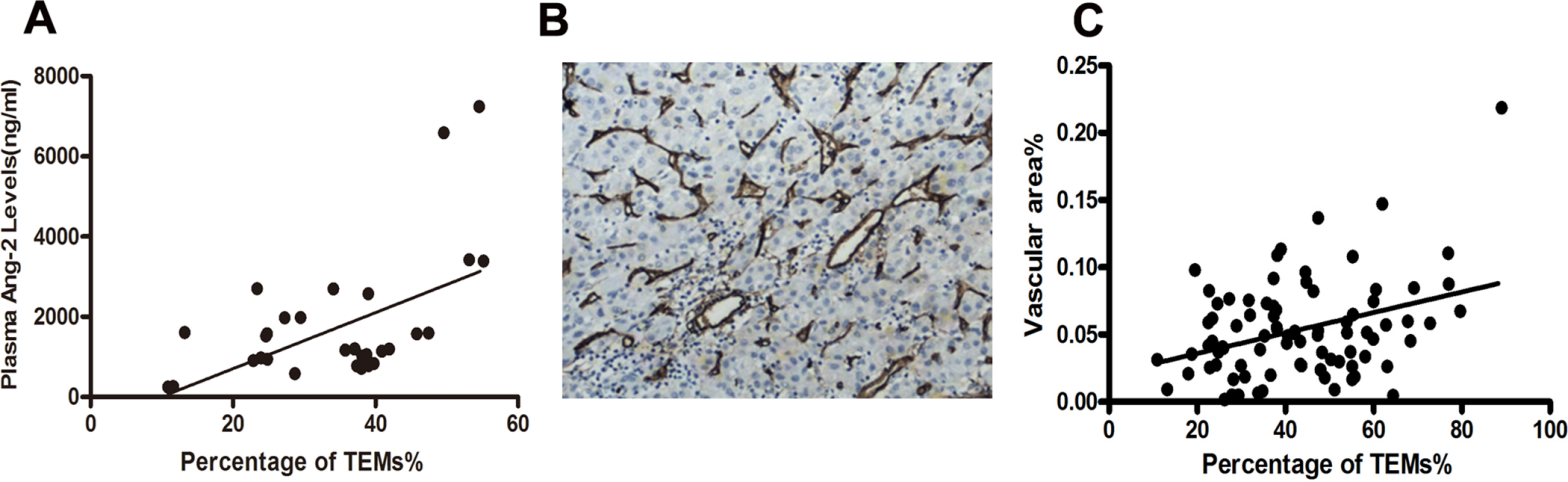
TEMs frequency in peripheral blood was correlated with Ang-2 concentration and microvessel area. (A) showed the positive correlation between percentage of TEMs and plasma Ang-2 concentration in HCC patients (n = 34, *P*<0.001, r^2^ = 0.294 by Pearson’s analysis). (B) Microvessels were represented by brownish yellow capillaries. Representative image is shown (100×). (C) showed the positive correlation between percentage of TEMs and vascular area as a percentage of tumor area (vascular area %) (*P*<0.001, r^2^ = 0.126).

The Ang-2 concentration in peripheral blood plasma of randomly selected 34 patients ranged from 243.7pg/ml to 7244.1pg/ml, which was positively correlated with the percentage of TEMs in peripheral blood monocytes (*P*<0.001, r^2^ = 0.294; Fig 3A).

### Vascular area/ tumor area in tumor tissues was positively corresponded with percentage of TEMs in peripheral blood monocytes

The median vascular area/ tumor area of all the enrolled 84 HCC patients was 4.93%. Fig 3B showed a representative piece of image of microvessel staining in tumor tissue. By analyzing with the percentage of TEMs in peripheral blood monocytes of HCC patients, there was a positive correlation between vascular area/ tumor area and TEMs percentage (*P*<0.001, r^2^ = 0.126; Fig 3C).

### TEMs distribution in tumor tissues

**Fig 4:**
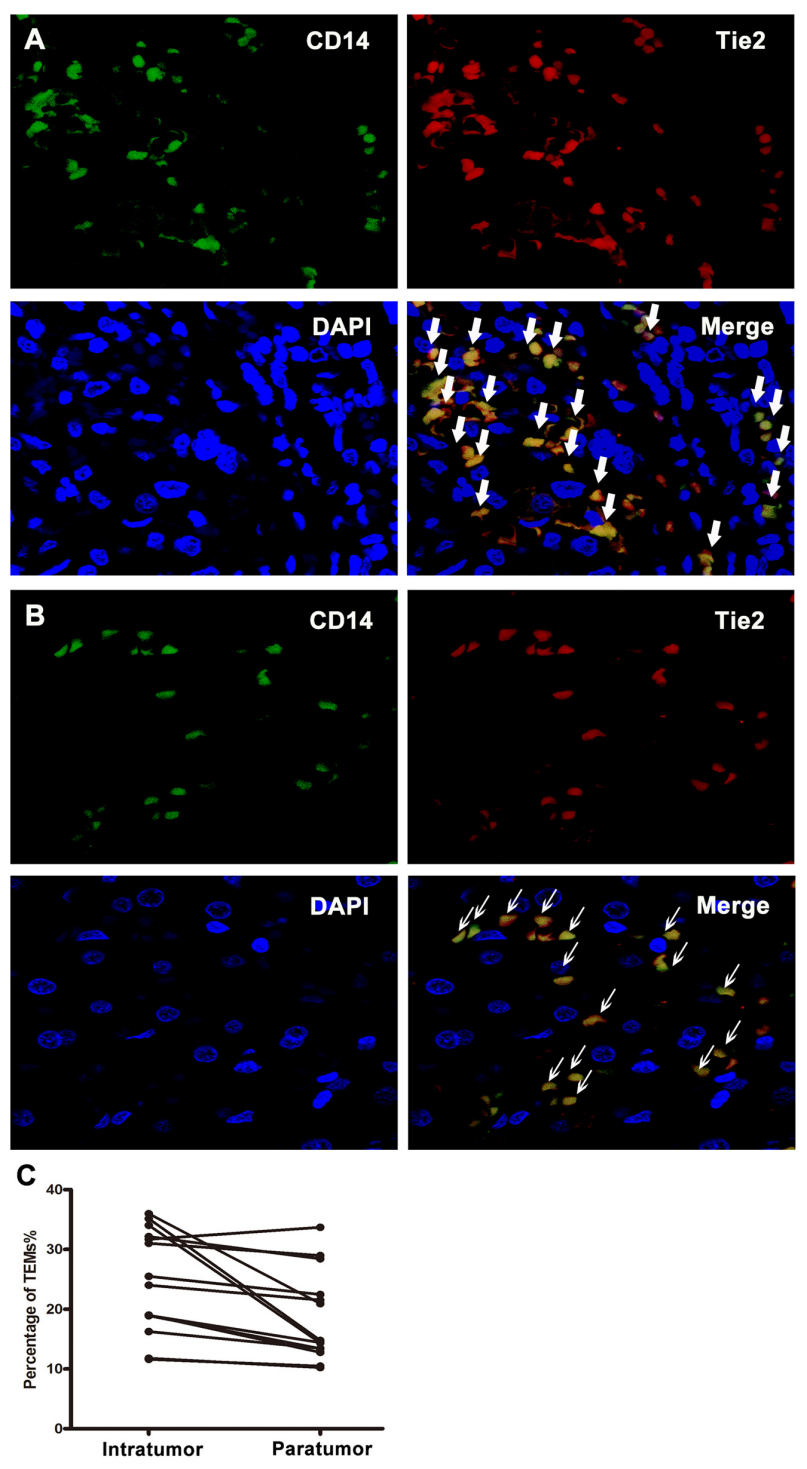
Distribution of TEMs by immunofluorescence staining and comparing TEMs intratumor with paratumor. (A) Monocytes surface marker CD14 stained with Alexa Fluor 488-labeled IgG (green), Tie2 with Alexa Fluor 594-conjugated IgG (red) and nuclei stained with dapi (blue). Big white arrows represent CD14^+^Tie2^+^ monocytes intratumor (1200×). (B) Monocytes surface marker CD14 stained with Alexa Fluor 488-labeled IgG (green), Tie2 with Alexa Fluor 594-conjugated IgG (red) and nuclei stained with dapi (blue). Thin white arrows represent CD14^+^Tie2^+^ monocytes paratumor (1200×). (C) displayed percentage of TEMs in intratumoral tissue was significantly higher than that in the corresponding paratumoral tissue (*P*<0.001).

Tumor and paratumoral tissues were randomly selected from 14 HCC patients to assess TEMs in the tumor microenvironment by calculating percentage of TEMs in infiltrating monocytes according to flow cytometry (Fig 4A and 4B). We found most TEMs had a trend of gathering together and distributed around vascular endothelial cells. The percentage of TEMs in tumor-infiltrating monocytes was significantly higher than that in the corresponding paratumor-infiltrating monocytes (*P*<0.001; Fig 4C).

### Prognostic significance of TEMs for HCC patients

**Fig 5:**
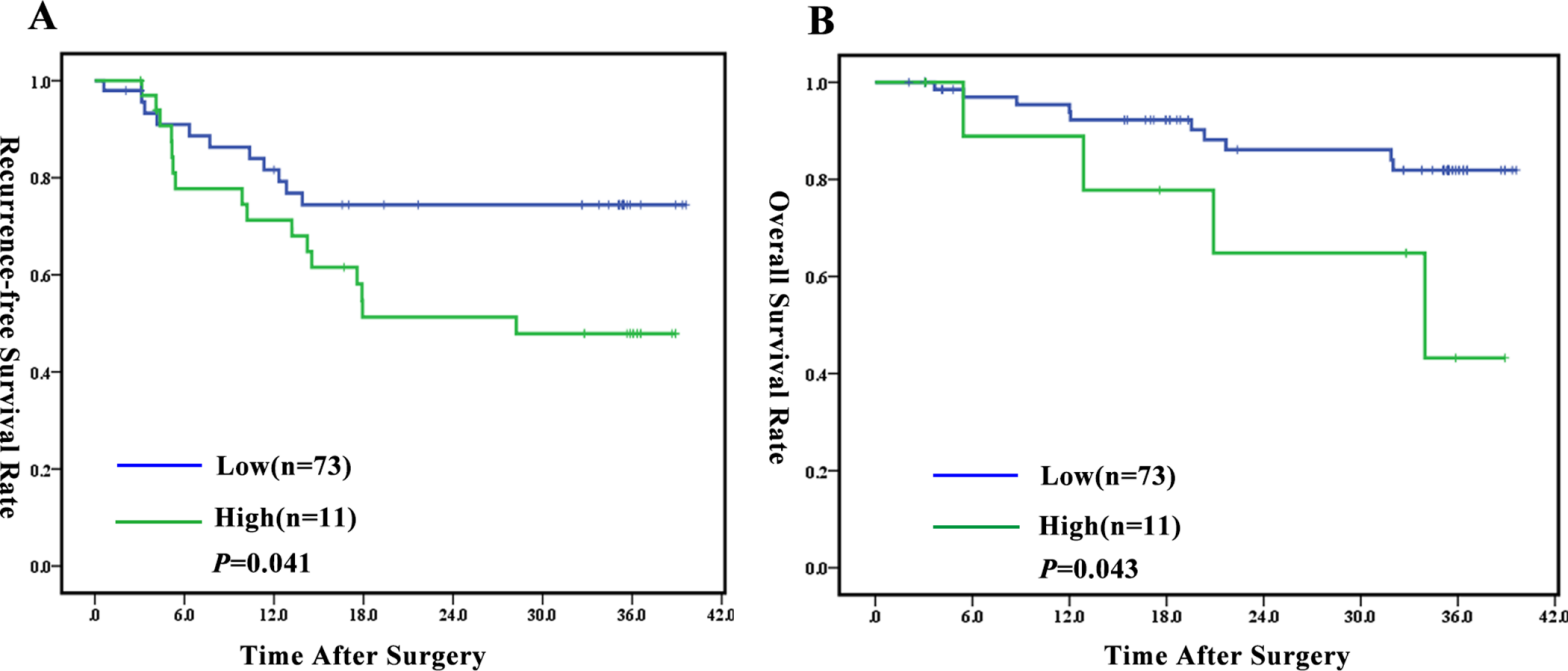
Recurrence-free and overall survival of HCC patients with different TEMs percentage in peripheral blood. Low percentage of TEMs in the peripheral blood was associated with better recurrence-free survival (A) and overall survival (B). The Log-Rank test was applied to compare between the groups.

Twenty-seven patients had tumor recurrence and 14 patients died by following up the 84 HCC patients post-operation for 32.7 months (median), ranged from 2.0 to 39.6 months, The 1-, 2-, 3-year OS rates were 92.4%, 84.5% and 75.8%, respectively and the corresponding RFS rates were 78.3%, 65.2% and 62.7%, respectively. In addition, univariate analysis revealed that presence of macroscopic tumor thrombi and the percentage of TEMs in peripheral blood monocytes were both associated with OS and RFS. Besides, serum AFP and vascular area/ tumor area were associated with OS, while tumor size was related with RFS (Table 2). The median RFS and OS were significantly poorer for patients with high percentage of TEMs than those with low percentage of TEMs (24.5 months vs. 31.5 months and 28.9 months vs. 35.7 months, respectively; *P* = 0.041 and *P* = 0.043, respectively; Fig 5A and 5B). In the multivariate analysis, the percentage of TEMs in peripheral blood monocytes (hazard ratio [HR] = 3.471, *P* = 0.039; HR = 2.524, *P* = 0.022) and macroscopic tumor thrombi (HR = 47.273, *P* = 0.002; HR = 21.457, *P* = 0.011) were independent risk factors for OS and RFS (Table 2).

**Table 2:** Univariate and multivariate analysis of clinicopathologic characteristics associated with survival and recurrence of 84 patients with HBV-related HCC.

Clinicopathologic characteristics	Overall survival			Recurrence-free survival		
	Uni-	Multivariate		Uni-	Multivariate	
	*P*	Hazard Ratio (95%CI)	*P*	*P*	Hazard Ratio (95%CI)	*P*
**Gender (male/female)**	0.980	-	NA	0.428	-	NA
**Median age (year)**	0.114	-	NA	0.064	-	NA
**Child-Pugh grade(A/B)**	0.428	-	NA	0.241	-	NA
**HBVDNA,Copies/ml (<10** ^**3**^ **/>10** ^**3**^ **)**	0.813	-	NA	0.405	-	NA
**Edmonson grading(I/II/III/IV)**	0.507	-	NA	0.723	-	NA
**Tumor size, cm (≤5/>5)**	0.055	-	NA	0.028	-	0.054
**Tumor number(single/multiple)**	0.352	-	NA	0.915	-	NA
**Microscopic Vascular invasion(present/absent)**	0.066	-	NA	0.660	-	NA
**AFP, ng/ml(≤20/>20)**	0.029	-	0.134	0.116	-	NA
**Macroscopic Vascular invasion(present/absent)**	0.000	47.273(4.153–538.048)	0.002	0.000	21.457(2.014–228.622)	0.011
**Vascular area/ tumor area**	0.023	-	0.716	0.135	-	NA
**BCLC stage (0/A/B/C)**	0.136	-	NA	0.549	-	NA
**Percentage of TEMs in peripheral blood monocytes**	0.043	3.471(1.067–11.291)	0.039	0.041	2.524(1.142–5.580)	0.022

Note: Kaplan-Meier survival estimates & Cox proportional hazards regression model for univariate & multivariate analysis. Statistically significant variables demonstrated at univariate analysis were adopted into Cox regression model.

## Discussion

TEMs are a subgroup of circulating and tumor-infiltrating myeloid cells with potent proangiogenic activity [5]. After their discovery in animal models and human tumor, TEMs have been considered as novel biomarkers and potential therapeutic targets. Whether TEMs frequency and function in HCC with HBV infection patients are different from the normal extent or associated with clinicopathological factors has not been evaluated previously. In this study, the results of IFS revealed that TEMs in HCC tissues had a trend of gathering together and distribute around vascular endothelial cells. The percentage of TEMs in tumor-infiltrating monocytes was significantly higher compared with that in paratumor-infiltrating monocytes (*P*<0.001). We also demonstrated that vascular area/ tumor area in tumor tissues was positively correlated with TEMs percentage in peripheral blood monocytes (*P*<0.001). These evidences may indicate the primary function of TEMs in tumor formation and development is derived from promoting tumor angiogenesis [5,14,15]. TEMs depletion could limit tumor growth, as well as increase the therapeutic effect of combretastatin A4 phosphate, which is the vascular disrupting agent [16].

The function of TEMs in tumor angiogenesis might be related to Ang-2/Tie2 signal pathway. TEMs could be recruited into tumor sites by Ang-2 which is an important chemokine for TEMs. Furthermore Ang-2 also stimulates Tie2 expression on the surface of monocytes [9]. Remarkably, our data demonstrated the percentage of TEMs in peripheral blood monocytes of HCC patients was positively correlated with plasma Ang-2 concentration (*P*<0.001, r^2^ = 0.294). Moreover, Forget et al [17] showed that TEMs could be recruited into tumor sites by macrophage colony-stimulating factor (M-CSF). The angiogenic function of TEMs in hypoxic tumor sites may be related to its secretion of VEGFA, MMP-9 and so on [5]. Coffelt et al [18] also demonstrated that Ang-2 meanwhile stimulates TEMs to express interleukin-10 and chemokine (C-C motif) ligand 17 (CCL17) in vitro. The cytokines may reduce T cells proliferation, increase the ratio of CD4^+^ T cells to CD8^+^ T cells, and accelerate the amplification of regulatory T cells (CD4^+^CD25^+^ FOXP3^+^).

Since the close connection between TEMs and HCC angiogenesis, the diagnostic value of TEMs for HCC with HBV-related liver cirrhosis was further investigated. As expected, the percentage of TEMs in peripheral blood monocytes of HCC patients was significantly increased compared with that in patients with HBV-related liver cirrhosis and healthy donors according to their surface marker (CD14, CD16 and Tie2). The sensitivity and specificity (76.2% and 76.2% between HCC and HBV-related cirrhosis, 81.0% and 60.9% between HCC patients and healthy donors) were figured out by ROC analysis. Despite its value in identifying HCC from liver cirrhosis and healthy donors, it should be noticed there was no statistical significance between HCC and benign liver tumor for the percentage of TEMs. Part of the reason was that 53.3% (8/15) of benign tumors consisted of hemangioma in our research, whose pathogenesis might be correlated with vasculogenesis and angiogenesis. It’s reported that Tie2 was up-regulated in hemangioma-derived endothelial cells [19].

In addition to the identification values, the elevated circulating TEMs may also play a crucial role in predicting prognosis of HCC patients. According to our observation, the percentage of TEMs in peripheral blood monocytes was a significant independent predictor of patients’ RFS and OS post-operation. Patients with more percentage of TEMs have 3.471 times higher risk of death and 2.524 times higher risk of recurrence post-operation than those with less percentage. These results suggested the increase in TEMs percentage was associated with HCC progression and might serve as a predictor of poor survival.

Matsubara et al [14] has reported TEMs increase in HCC patients with hepatitis C virus. Compared with their research, each HCC patient in our study was exclusively with HBV infection. Furthermore we demonstrated TEMs frequency in the peripheral blood could be utilized to anticipate the prognosis after hepatectomy. It’s a useful supplement to their work. In the next step, it would be meaningful to study whether TEMs could be applied as useful markers to display clinical responses of patients receiving anti-cancer chemotherapy or antiangiogenic drugs [20]. The identification of cytokines by TEMs and the surrounding factors regulating TEMs recruitment or function may produce novel anticancer therapies. A recent study has shown genetic modified monocytes as delivery vehicles which infiltrated breast cancer for therapeutic purpose [21], so it’s also a promising approach to utilize TEMs as gene-delivery vehicles for HCC treatment in the future.
